# Acute radiation impacts contractility of guinea-pig bladder strips affecting mucosal-detrusor interactions

**DOI:** 10.1371/journal.pone.0193923

**Published:** 2018-03-07

**Authors:** Bronagh M. McDonnell, Paul J. Buchanan, Kevin M. Prise, Karen D. McCloskey

**Affiliations:** Centre for Cancer Research and Cell Biology, School of Medicine, Dentistry and Biomedical Sciences, Queen’s University Belfast, Belfast, Northern Ireland, United Kingdom; Northwestern University Feinberg School of Medicine, UNITED STATES

## Abstract

Radiation-induced bladder toxicity is associated with radiation therapy for pelvic malignancies, arising from unavoidable irradiation of neighbouring normal bladder tissue. This study aimed to investigate the acute impact of ionizing radiation on the contractility of bladder strips and identify the radiation-sensitivity of the mucosa vs the detrusor. Guinea-pig bladder strips (intact or mucosa-free) received *ex vivo* sham or 20Gy irradiation and were studied with *in vitro* myography, electrical field stimulation and Ca^2+^-fluorescence imaging. Frequency-dependent, neurogenic contractions in intact strips were reduced by irradiation across the force-frequency graph. The radiation-difference persisted in atropine (1μM); subsequent addition of PPADs (100μM) blocked the radiation effect at higher stimulation frequencies and decreased the force-frequency plot. Conversely, neurogenic contractions in mucosa-free strips were radiation-insensitive. Radiation did not affect agonist-evoked contractions (1μM carbachol, 5mM ATP) in intact or mucosa-free strips. Interestingly, agonist-evoked contractions were larger in irradiated mucosa-free strips vs irradiated intact strips suggesting that radiation may have unmasked an inhibitory mucosal element. Spontaneous activity was larger in control intact vs mucosa-free preparations; this difference was absent in irradiated strips. Spontaneous Ca^2+^-transients in smooth muscle cells within tissue preparations were reduced by radiation. Radiation affected neurogenic and agonist-evoked bladder contractions and also reduced Ca^2+^-signalling events in smooth muscle cells when the mucosal layer was present. Radiation eliminated a positive modulatory effect on spontaneous activity by the mucosa layer. Overall, the findings suggest that radiation impairs contractility via mucosal regulatory mechanisms independent of the development of radiation cystitis.

## Introduction

Radiation-induced bladder toxicity (RIBT) is a common and poorly managed clinical condition which negatively impacts the health of patients undergoing radiation therapy for pelvic malignancies [1,2]. RIBT is an unavoidable consequence resulting from collateral damage to normal tissues located close to the tumour site, limiting the effective dose of radiotherapy that can be given; in practice, a maximum dose of 65-72Gy is delivered in fractionated dose regimens of 2Gy [3]. RIBT involves physiological and structural changes to bladder cells resulting from direct irradiation, bystander responses from irradiated target cells to neighbouring non-irradiated cells and visceral organ cross-talk [4].

Acute RIBT affects 23–80% of patients, manifested as lower urinary tract symptoms (LUTS) including urgency, dysuria, frequency and haematuria [3]. Bladder storage capacity typically decreases during successive radiotherapy sessions [5] with concomitant development of LUTS. These acute symptoms may be temporary, reducing in severity after several weeks/months and are conservatively managed with lifestyle changes or anti-muscarinic drugs. A proportion of patients develop late RIBT characterized by irreversible organ fibrosis, limited reservoir capacity and may require surgical intervention including bladder augmentation, urinary diversion or complete cystectomy [6–9].

Radiation damage occurs in cells of the bladder wall in animal models and patients such that the normal physiological barrier function of the urothelium is impaired, exposing underlying nerves, interstitial cells, smooth muscle, and the microvasculature to urine contents which has an irritative effect on detrusor smooth muscle [10]. Significant urothelial damage persists several months after therapy cessation, consistent with slow turnover rate of the urothelium [11]. Radiation-induced morphological damage to nerves and smooth muscle includes oedema and necrosis progressing to replacement of smooth muscle with fibroblasts and increased collagen deposition [12]. These late cellular changes accompany bladder dysfunction ultimately resulting in reduced compliance, reduced capacity and a high-pressure bladder [13,14].

Our knowledge of the impact of radiation on bladder contractility and subsequent changes to physiological mechanisms is limited. Early studies demonstrated an immediate tetrodotoxin-resistant, contractile response in excised bladder strips during irradiation which either developed slowly or exhibited tachyphylaxis [15]. While this sub-acute contraction may not reflect acute RIBT symptoms in patients or animal models, it is clear that radiation directly impacts bladder contractility. Given that in healthy bladder, detrusor smooth muscle activity is influenced by interaction with the adjacent mucosa and this mucosal input appears to be enhanced in bladder dysfunction [16], it is important to determine whether radiation damage occurs in the mucosa and/or detrusor. Importantly, we do not yet know whether radiation impacts bladder contractility independent of urothelial damage and inflammation.

The present study tested our hypothesis that ‘*ex vivo irradiation directly affects bladder strip contractility through targeting mucosal modulation of detrusor contraction’*. Experiments were designed to compare neurogenic, agonist-evoked and spontaneous contractions and Ca^2+^-signalling in non-irradiated and irradiated bladder strips. The findings demonstrate that irradiation reduced neurogenic contractions when the mucosal layer was present through a purinergic-sensitive mechanism. Cholinergic and purinergic evoked contractions were larger in irradiated mucosa-free strips indicating possible loss of an inhibitory mucosal component. Finally, irradiation eliminated mucosal-modulation of spontaneous contractions and spontaneous Ca^2+^-signalling was reduced, showing effects on bladder smooth muscle physiology.

## Materials and Methods

### Tissue preparation

Bladders were removed from male Dunkin Hartley guinea-pigs (250-500g; 5–8 weeks) that had been killed by cervical dislocation in accordance with Schedule 1 (UK Animal Scientific Procedures Act) and with institutional AWERB (Animal Welfare Ethics Research Board) committee approval. Female animals were not included as we wanted to exclude the effect of oestrogen levels on bladder contractility during the oestrous cycle which is known to affect the parameters being measured in the present study [17]. Bladders were opened with a longitudinal incision and 4 strips from the body region (10mm x 2mm x 2mm) were prepared as intact or detrusor strips after removal of the mucosal layer by sharp dissection in cold Krebs’ solution. Mucosa removal was confirmed when the detrusor surface was visible, indicated by lack of major vasculature and was validated by fixation in 10% neutral buffered formalin and processing for haematoxylin and eosin histology (N = 1).

### Radiation protocols

A single dose 20Gy, 225 kV at a dose rate of 0.52Gy/min was delivered to bladder strips, pinned to a dissection dish with minimal stretch in Krebs’ solution using an X-Rad 225 generator (Precision X-ray Inc, Connecticut, USA). Non-irradiated control strips were prepared in an identical fashion but the X-ray machine was not switched on. Single doses of 20Gy are commonly used in rodent *in* vivo and *ex vivo* tissue irradiation studies of radiation-induced bladder damage; 20Gy has been determined as the ED_50_ in studies measuring functional outputs including reduced bladder capacity [18–20].

### In vitro myography

Tissue strips were mounted in organ baths within 30 minutes of irradiation or sham irradiation which occurred typically 45–60 minutes after sacrifice, between 2 stimulating electrodes to an initial tension of 10mN and allowed to equilibrate for 1hr, during which time spontaneous contractions typically developed. Tissues were perfused with oxygenated Krebs’ solution (37°C) and drugs applied via the perfusion system (1-2ml/min). Electrical field stimulation (EFS) was used to generate neurogenic contractions (0.3ms pulse width, 70V, 10s duration, 0.5-16Hz). Data was acquired by a computer running Chart software (University of Strathclyde, UK). The experimental setup enabled myography to be performed on 4 strips simultaneously. The majority of protocols comprised 2 control and 2 irradiated strips, all intact or all mucosa-free. In a number of experiments, the 4 strips were; control intact, control mucosa-free, irradiated intact and irradiated mucosa-free. The standard control for strip viability was the contractile response to 60mM K^+^ Krebs’ solution (e quimolar substitution of NaCl with KCl).

### Data analysis

Spontaneous contractions were measured off-line over an analysis window of 5 min and are presented as contraction integral (area under curve, AUC, g.min), amplitude (measured in g and expressed as tension, mN) and frequency (events/min). A threshold of 10% of the maximal amplitude was set; the amplitude and frequency of spontaneous contractions exceeding the threshold was calculated during the analysis window. Neurogenic contractions were measured as amplitude and agonist-evoked contractions as AUC (first 5 min of carbachol and high-K^+^ exposure and first 3 min of ATP exposure). Data were analysed using Clampfit (pClamp, v10.3), Microsoft Excel and Prism (Graphpad v5.0) software and are expressed as mean and standard deviations (SD) with N and n referring to the number of animals and strip preparations respectively. Unpaired t-tests, 1-way ANOVA or 2-way repeated measures ANOVA with Bonferroni post-hoc tests were used with p<0.05 considered as significant.

### Fluorescence imaging

Tissue preparations containing urothelium, lamina propria and smooth muscle bundles were loaded with the Ca^2+^-indicator, fluo-4AM (1μM). Preparations were imaged with an EMCCD camera (Nikon, London, UK) and images acquired at 20fps with WinFluor software (University of Strathclyde, Glasgow, UK). Changes in fluorescence intensity (ΔF/F_0_) were calculated off-line by normalising the background-corrected fluorescence (F) to baseline (F_0_) which was obtained by averaging 150 frames during quiescent periods in active cells. Data analysis and statistical tests were performed with Microsoft Excel, Prism (Graphpad Prism, v5.02) and Clampfit (pClamp, v10.3). The amplitude, frequency and area under the curve (AUC) of spontaneous Ca^2+^ events were measured during 120s of continuous recording. Peak fluorescence intensity occurring during the recording window was noted; events with fluorescence intensities greater than 10% of the peak were accepted for analysis. Amplitude was measured as fluorescence intensity (ΔF/F_0_); event duration was measured when ΔF/F_0_ exceeded 10% of the peak amplitude and recovered to this level and frequency was calculated as number of events/min. AUC is reported as ΔF/F_0_.min. Unpaired t-tests were used with p<0.05 considered significant.

### Drugs and solutions

Krebs’ solution used for in vitro myography experiments comprised (mM): NaCl (120), KCl (5.9), NaHCO_3_ (25), NaH_2_PO_4_ (0.33), glucose (5.5), CaCl_2_ (2.5),1.2 MgCl_2_ and was constantly gassed with 95% O_2_/5% CO_2_. HEPES-buffered Krebs’ solution used in Ca^2+^-imaging experiments comprised: NaCl (125), KCl (5.36), glucose (11), HEPES (10), KH_2_PO_4_ (0.44), NaH_2_PO_4_ (0.33), MgCl_2_ (1) CaCl_2_ (1.8), pH was adjusted to 7.4 with NaOH.

Drugs were prepared as stock solutions and diluted to their final concentration in Krebs’ solution so that the vehicle concentration was <0.1%; atropine sulphate; PPADs, carbachol and ATP were purchased from Sigma-Aldrich (UK).

## Results

Contractile responses were compared between non-irradiated and irradiated bladder strips with the mucosal layer intact (*intact*) and the mucosa removed (*mucosa-free*). This was to establish: (1) if radiation affected contractile responses in intact or mucosa-free strips; (2) if the mucosal layer modified the contractile responses of the detrusor layer and (3) if this relationship was changed after irradiation.

### Irradiation impacted neurogenic contractions

**Fig 1:**
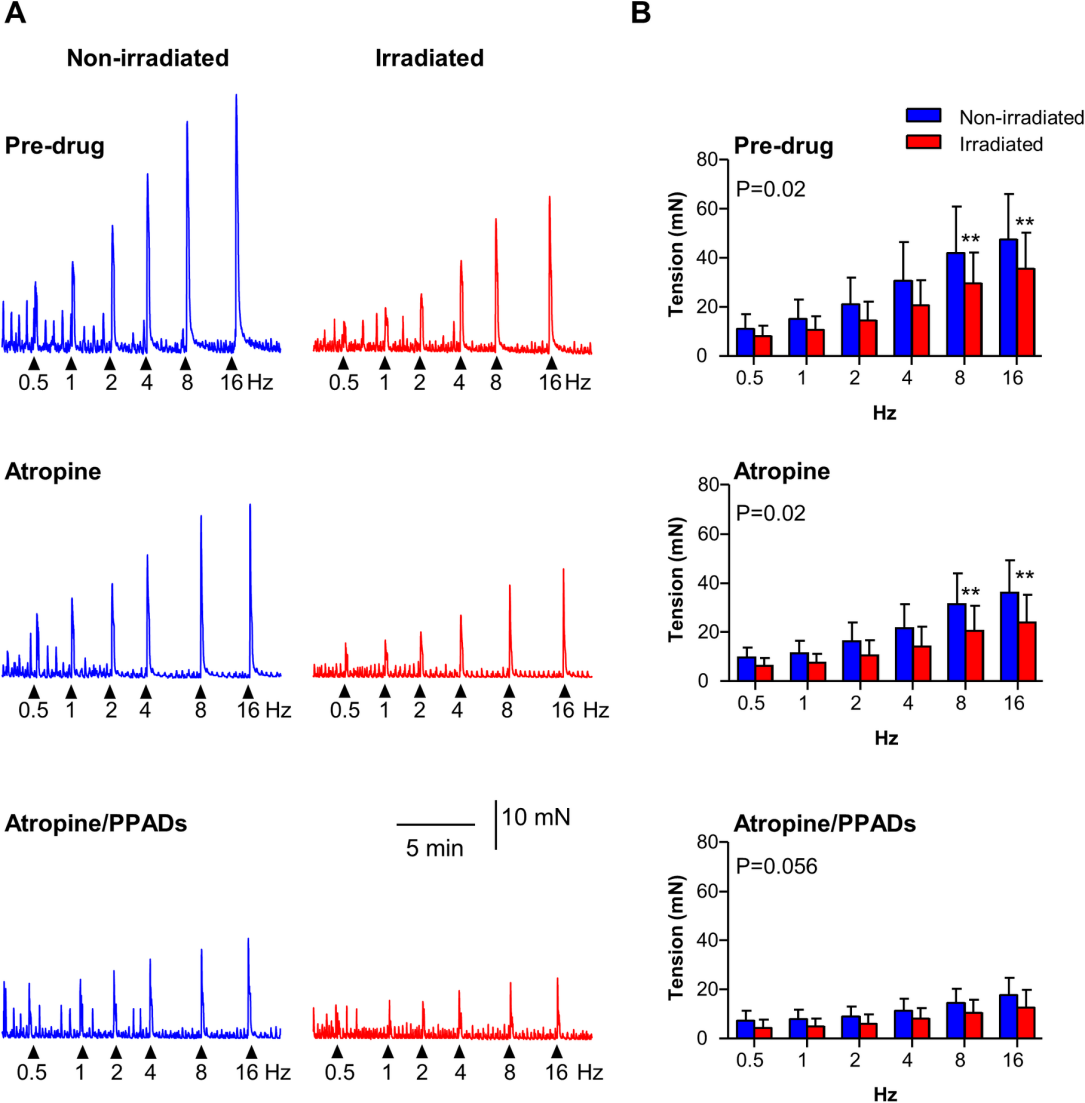
Neurogenic contractions in intact strips are reduced by irradiation. (A) Original traces of EFS-evoked neurogenic contractions in non-irradiated controls (blue) and 20Gy irradiated intact strips (red). Atropine (1μM) reduced neurogenic contractions at all frequencies and subsequent addition of PPADs (100μM) caused further reduction. (B) Summary graphs (mean ± SD) showing that neurogenic contractions in irradiated strips (N = 12, n = 19) were significantly smaller than non-irradiated controls across the force-frequency range (P = 0.02, N = 12, n = 18) and at 8Hz and 16Hz (P<0.05, post-hoc test). Atropine did not affect the radiation-induced significant difference seen between non-irradiated (N = 9, n = 13) and irradiated strips (N = 9, n = 14). PPADs eliminated the radiation-induced effect at 8Hz and 16Hz (N = 9, n = 13; N = 9, n = 14). ** denotes P<0.01 vs. non-irradiated, 2-way repeated measures ANOVA, Bonferroni post-hoc test.

Neurogenic contractions were evoked by EFS in non-irradiated (N = 12, n = 18) and irradiated intact strips (N = 12, n = 19) (Fig 1A). The force-frequency contraction curve (Fig 1B) was reduced by irradiation (P = 0.02) and significance detected at higher frequencies in post-hoc tests (8Hz and 16Hz, P<0.01). The pan-muscarinic receptor antagonist atropine (1μM) did not affect radiation-differences observed at 8Hz and 16Hz between non-irradiated (N = 9, n = 13) and irradiated strips (N = 9, n = 14); these persisted across the frequency-response range (P = 0.02). Subsequent addition of the pan-purinergic receptor antagonist, PPADs (100μM), eliminated the radiation-effect across the frequency-response range (P = 0.056) and at 8Hz/16Hz. The significant effects of atropine and PPADs on bladder neurogenic contractions are well established and are not further analysed here.

**Fig 2:**
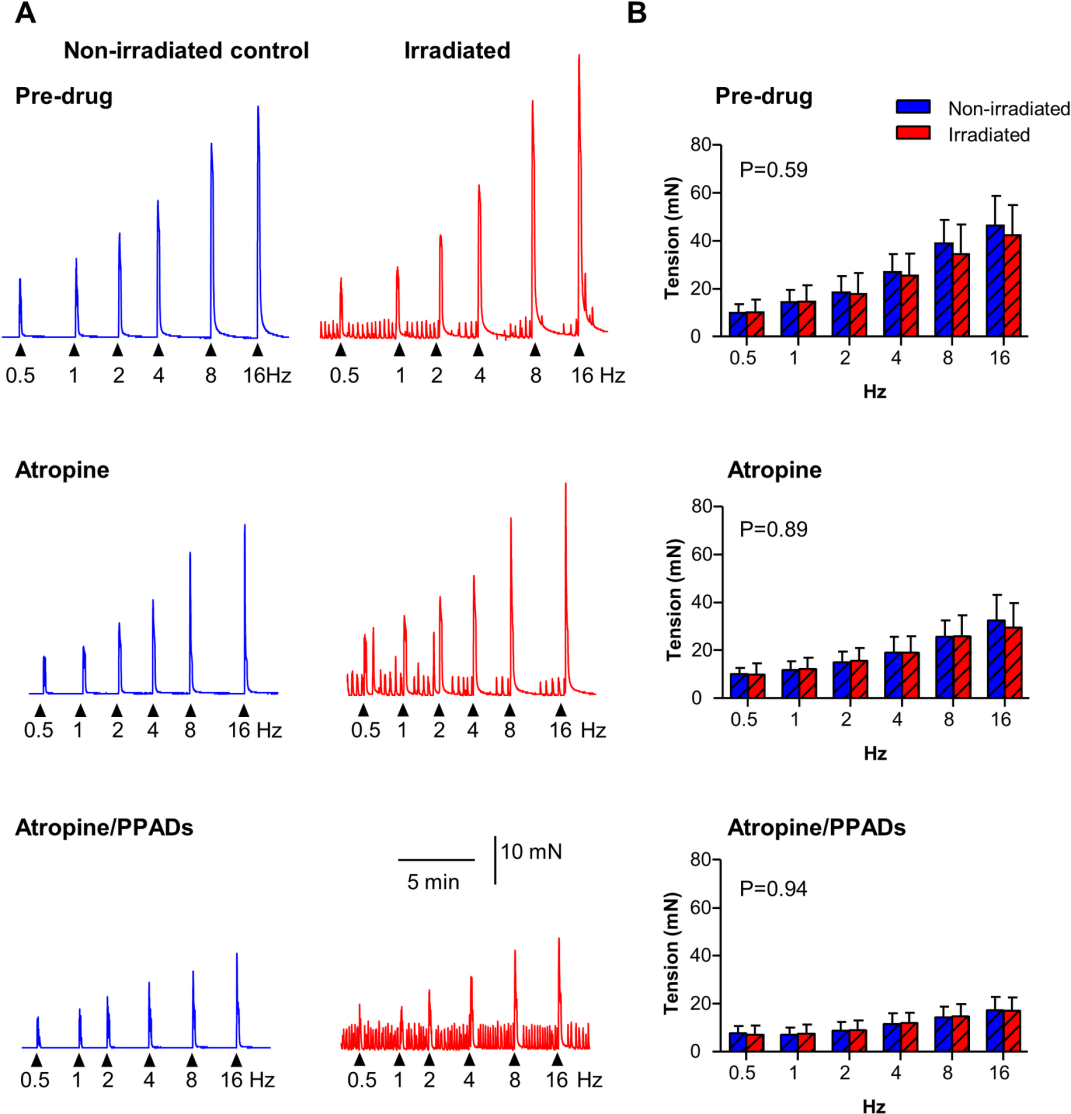
Neurogenic contractions in mucosa-free strips are not affected by irradiation. (A) Original traces of EFS-evoked neurogenic contractions in non-irradiated controls (blue) and 20Gy irradiated mucosa-free strips (red). Atropine (1μM) reduced neurogenic contractions at all frequencies and subsequent addition of PPADs (100μM) caused further reduction. (B) Summary graphs (mean ± SD) showing that neurogenic contractions in irradiated strips (N = 6, n = 12) were not different than non-irradiated controls (N = 6, n = 12) across the force-frequency range (P = 0.59) and at each frequency tested (post-hoc test). Atropine (N = 6, n = 12) and subsequent addition of PPADs (N = 6, n = 12) affected both non-irradiated and irradiated strips similarly 2-way repeated measures ANOVA, Bonferroni post-hoc test. * denotes P<0.05.

Similar protocols on mucosa-free strips (Fig 2A and 2B) which were irradiated after removal of the mucosal layer, showed that the force-frequency contraction curves were not affected by irradiation (pre-drug P = 0.59, atropine P = 0.89, atropine/PPADs P = 0.94, all N = 6, n = 12).

**Fig 3:**
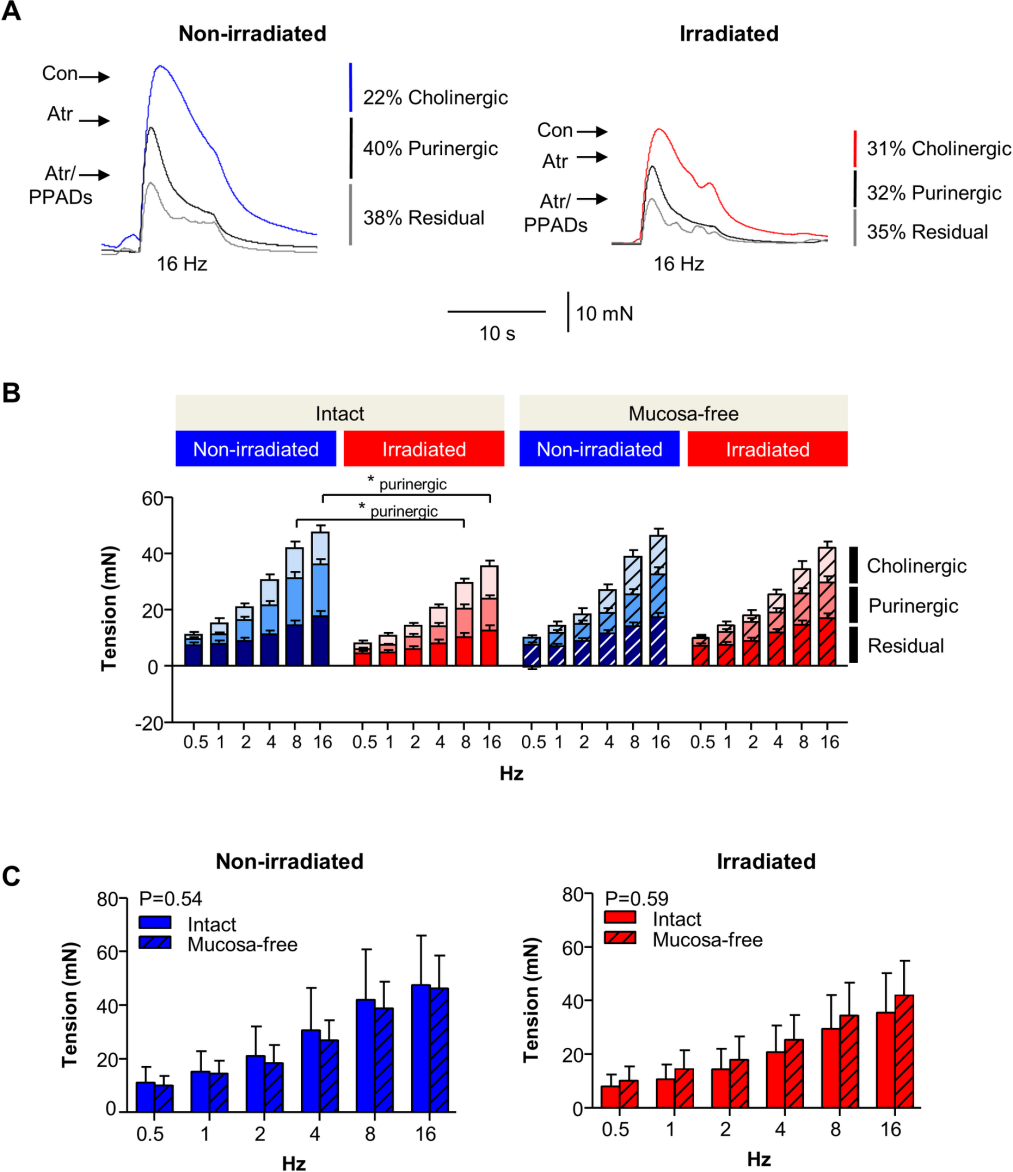
Comparison of cholinergic, purinergic and residual neurogenic contractions in non-irradiated and irradiated bladder strips. (A) Traces of neurogenic contractions (16Hz) on an expanded timescale from Fig 1 showing cholinergic, purinergic and residual components. Control (Con), atropine (Atr), Atr/PPADs (atropine and PPADs present). (B) Cholinergic, purinergic and residual components of neurogenic contractions were calculated from atropine-sensitive, PPADs-sensitive and atropine/PPADs-resistant neurogenic contractions in irradiated and non-irradiated, intact and mucosa-free bladder strips. The purinergic component of neurogenic contractions was reduced in irradiated intact strips, compared to non-irradiated (P<0.05). * denotes P<0.05. (C) Bar charts from data in Figs 1 and 2 showing that absence of the mucosal layer *per se* had no significant effect on the amplitude of neurogenic contractions in control tissues (P = 0.54, 2-way ANOVA with Bonferroni post-hoc test). Similar findings were obtained with irradiated tissues (P = 0.59).

Analysis of cholinergic (control minus atropine-resistant contractions), purinergic (atropine minus atropine/PPADs-resistant contractions) and residual (drug-resistant) components of neurogenic contractions confirmed that irradiation significantly reduced the purinergic (PPADS-sensitive) component of neurogenic contractions in intact tissues (Fig 3A and 3B). Traces obtained at 16Hz in all conditions are shown on an expanded scale to illustrate cholinergic, purinergic and residual components.

Interestingly, the irradiation effect observed on neurogenic contractions in intact strips was not mimicked by physical removal of the mucosal layer. Frequency-contraction curves were similar between intact and mucosa-free strips whether non-irradiated (P = 0.54) or irradiated (P = 0.59, Fig 3C).

### Agonist-evoked contractions were affected by irradiation

**Fig 4:**
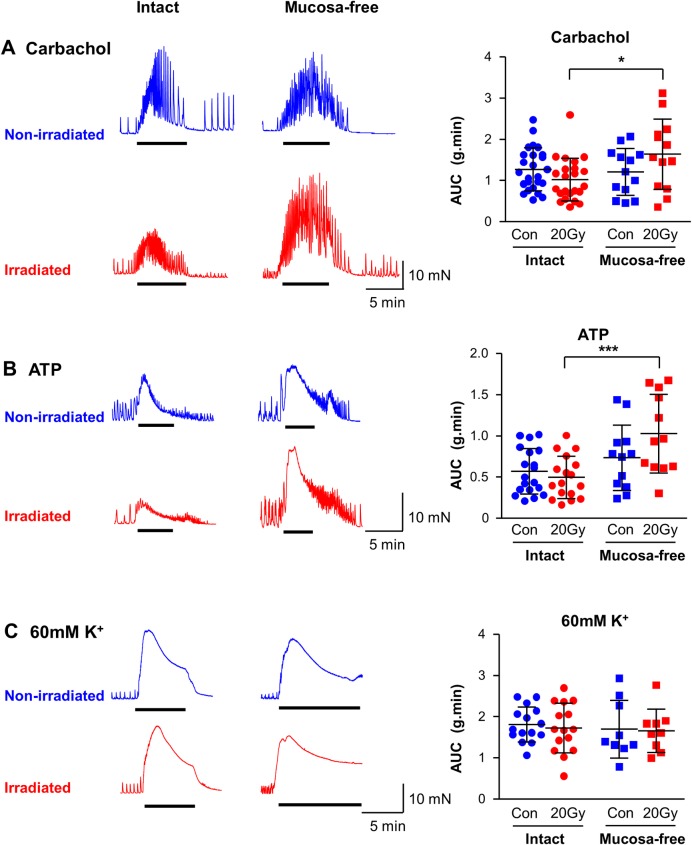
Irradiation and contractions evoked by carbachol, ATP and K^+^. (A) Representative carbachol (1μM) contractions in non-irradiated (blue) and 20Gy-irradiated (red), intact or mucosa-free bladder strips. Summary bar chart (mean ± SD) of force integral (AUC) showing that radiation did not affect carbachol-contractions in intact or mucosa-free strips. Interestingly, in irradiated strips, the mucosal layer apparently suppressed carbachol-contractions. * denotes P<0.05, unpaired *t*-test. Intact: non-irradiated, N = 17, n = 25; irradiated, N = 17, n = 23. Mucosa-free: non-irradiated, N = 7, n = 13; irradiated, N = 7, n = 13. (B) Representative ATP (5mM) contractions. Summary AUC bar chart shows ATP-contractions were unaffected by irradiation in intact or mucosa-free bladder strips. In irradiated strips, the mucosal layer apparently suppressed contractions (P<0.001). ***denotes P<0.001. Unpaired *t*-test. Intact: non-irradiated, N = 13, n = 19; irradiated, N = 13, n = 17. Mucosa-free: non-irradiated, N = 6, n = 12; irradiated, N = 6, n = 12. (C) Representative 60mM K^+^ contractions. Summary AUC bar chart shows these contractions were unaffected by irradiation in intact or mucosa-free strips. In contrast to carbachol and ATP, the mucosal layer had no impact on 60mM K^+^ contractions. Intact: non-irradiated, N = 12, n = 15; irradiated, N = 12, n = 15. Mucosa-free: non-irradiated, N = 5, n = 9; irradiated, N = 5, n = 9.

The major excitatory neurotransmitters in guinea-pig bladder are acetylcholine and ATP, released from parasympathetic nerves and potentially non-neuronal sources (urothelial or interstitial cells). To investigate whether the radiation-effect on EFS-contractions was a pre- or post-synaptic phenomenon, carbachol and ATP were tested in control and irradiated strips (Fig 4).

Carbachol-evoked contractions (1μM), comprising phasic activity superimposed on a tonic contraction, were similar in intact irradiated and non-irradiated strips (N = 17, n = 25, non-irradiated vs irradiated N = 17, n = 23, Fig 4A, P>0.05). Similarly, in mucosa-free strips, irradiation did not affect carbachol contractions (both N = 7, n = 13, P>0.05). Interestingly, carbachol contractions in irradiated mucosa-free strips (N = 7, n = 13) were larger than in irradiated intact strips (N = 17, n = 23; P<0.01) yet, removal of the mucosal layer in non-irradiated strips did not mimic the radiation-effect (P>0.05.)

Similarly, irradiation did not affect ATP-evoked contractions (5mM [21,22]) in intact (both N = 13, n = 19 non-irradiated, n = 17, irradiated) or mucosa-free strips (both N = 6, n = 12; P>0.05, Fig 4B). Absence of the mucosal layer had little effect on ATP-evoked contractions in non-irradiated strips but was associated with larger contractions in irradiated tissues (P<0.001). These findings suggest that in intact preparations, the effect of radiation on detrusor agonist-evoked contraction is masked or counteracted by the mucosa.

To test whether the ability of bladder smooth muscle to contract was affected by irradiation, tissues were challenged with 60mM K^+^ (high-K^+^) to evoke contractions in the absence of receptor-mediated stimulation (Fig 4C). Notably, in both intact (N = 12, n = 15) and mucosa-free tissues (N = 5, n = 9), high-K^+^ contractions were unaffected by irradiation (P>0.05) indicating that the intracellular contractile mechanisms of bladder smooth muscle were not damaged by radiation. High-K^+^ contractions (AUC) were also not affected by mucosal layer removal (P>0.05).

### Spontaneous activity and irradiation

**Fig 5:**
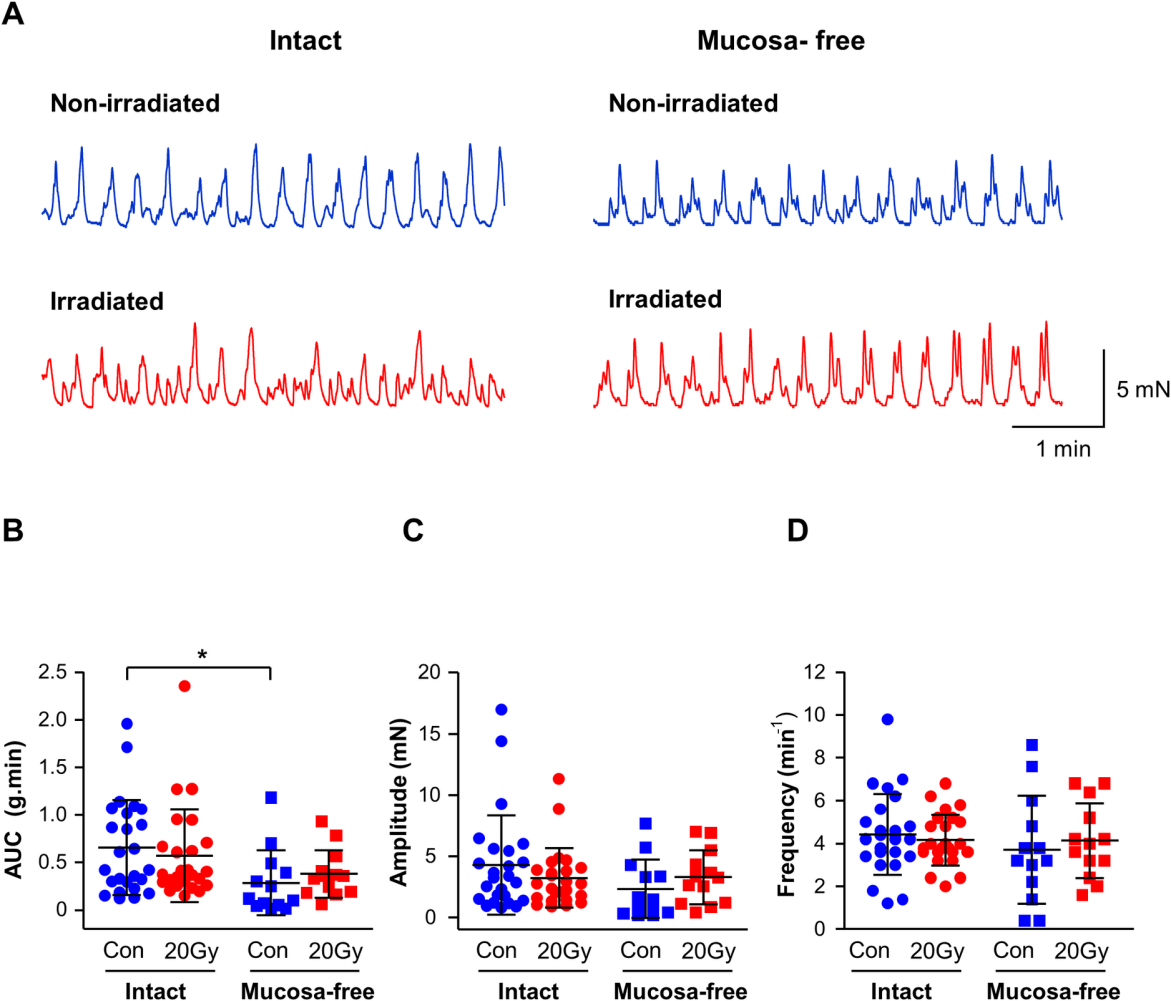
Spontaneous activity and *ex-vivo* irradiation. (A) Representative traces of spontaneous activity from non-irradiated (blue) and irradiated (red) bladder strips which were either intact, or mucosa-free. (B) AUC (mean ± SD) of spontaneous activity (g.min) was unaffected by irradiation (20Gy) (blue vs. red) in intact or mucosa-free bladder strips. The intact mucosal layer enhanced activity in non-irradiated strips (P<0.05, unpaired *t*-test) however this effect was lost in irradiated strips. * denotes P<0.05. (C) Irradiation did not affect the amplitude (mean ± SD) of spontaneous contractions in intact or mucosa-free bladder strips. The amplitude of contractions was also similar between intact and mucosa-free, non-irradiated and irradiated strips. (D) The frequency (mean ± SD) of spontaneous contractions was similar in all bladder strips. * denotes P<0.05. Intact: non-irradiated, N = 17, n = 25; irradiated, N = 17, n = 25. Mucosa-free: non-irradiated, N = 7, n = 13; irradiated, N = 7, n = 13.

Guinea-pig bladder strips typically exhibited spontaneous contractions that were maintained up to 4hr during experiments, and occurred in both intact and mucosa-free strips (Fig 5A). Radiation did not significantly affect spontaneous activity (AUC), amplitude or frequency in intact strips (both N = 17, n = 25, P>0.05, Fig 5B–5D) or mucosa-free strips (both N = 7, n = 13). Consistent with other studies [21] spontaneous activity (AUC) in intact, non-irradiated strips (N = 17, n = 25) was larger than in mucosa-free strips (N = 7, n = 13, P<0.05) although phasic contraction amplitude and frequency were similar (P>0.05, Fig 5B–5D). This observation was absent in irradiated strips, suggesting that irradiation eliminated a mucosal-modulatory effect on detrusor spontaneous contractions.

### Calcium signalling in smooth muscle was reduced by irradiation

**Fig 6:**
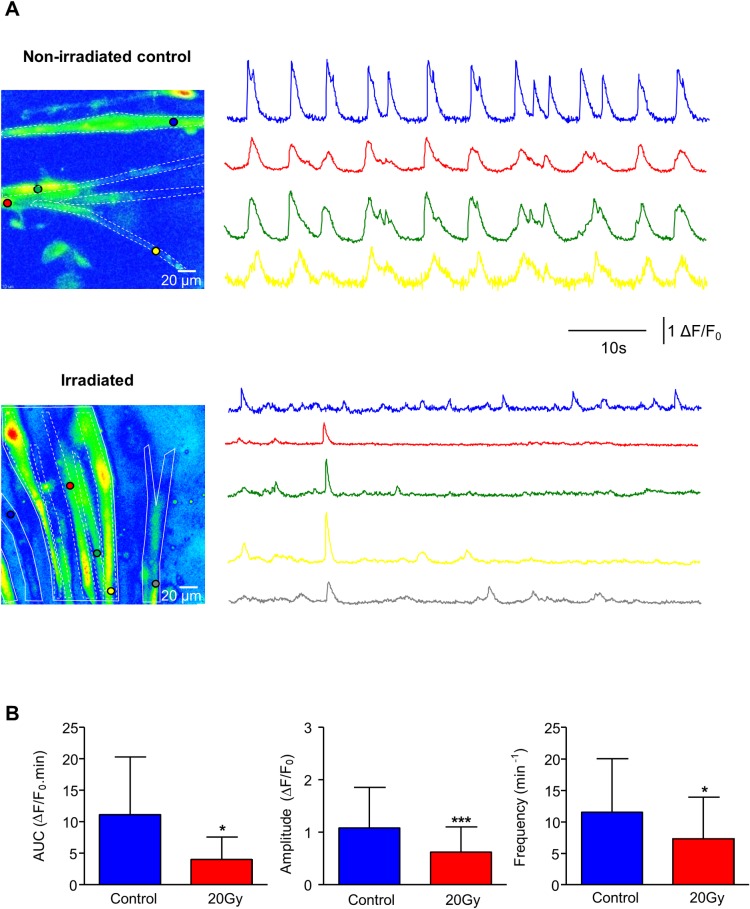
Irradiation and Ca^2+^-signalling in ex vivo bladder tissue. (A) Micrographs of fluo-4 loaded preparations. Regions of interest in cells within the field of view are indicted by coloured circles. The corresponding traces of Ca^2+^-transients in each coloured region of interest are shown as intensity-time plots. Micrographs and traces are shown for control and irradiated bladder tissues. (B) Spontaneous Ca^2+^-activity in irradiated smooth muscle cells measured as AUC was significantly smaller than in non-irradiated controls (p<0.05). Amplitude and frequency of spontaneous Ca^2+^-transients in irradiated smooth muscle cells were less than in non-irradiated controls (p<0.001, p<0.05 respectively). Non-irradiated controls: n = 411 events in n = 23 cells, N = 4; Irradiated: n = 412 cells in n = 28 cells, N = 4). * denotes P<0.05, *** denotes P<0.001, unpaired *t-*test).

Having established a negative radiation effect on neurogenic and receptor-mediated contractions via the mucosa, and elimination of mucosal-modulation of spontaneous contractions, spontaneous Ca^2+^ signalling was then investigated in irradiated smooth muscle cells (SMC) within mucosal preparations, similar to Gray et al [23]. Cell types within mucosal preparations were distinguishable by their distinctive morphologies viewed by changing the focal plane at the area of interest. SMC fired regular spontaneous Ca^2+^-transients which appeared to be somewhat synchronized with neighbouring SMC (Fig 6A). Ca^2+^-transient amplitudes were notably smaller in irradiated preparations and appeared to be less synchronous. Overall activity, (AUC) was reduced in irradiated (N = 4, n = 28 cells) compared to non-irradiated SMC (N = 4, n = 23 cells, P<0.05); this reduction was reflected by lower amplitude (P<0.001), less frequent (7.4±1.2 min^-1^ vs. 11.6±1.8 min^-1^; P<0.05) activity (n = 411 events in 23 cells from non-irradiated tissues vs n = 412 events in 28 cells from irradiated tissues, Fig 6B).

## Discussion

The present study was designed to assess irradiation impact on the contractile properties of bladder tissue and has demonstrated negative effects on neurogenic contractions, complex effects on agonist-evoked contractions and reduced SMC Ca^2+^-signalling. A single dose of 20Gy was used as *in vivo* studies of RIBT commonly use this protocol which has been determined as the ED_50_ in studies measuring functional outputs such as reduced bladder capacity [14,18–20]. Here, we aimed to determine how *ex vivo* irradiation affected contractile responses of bladder strips, independent of the establishment of inflammatory mechanisms that underpin radiation cystitis and damage to the urothelium. In all contractility experimental series, parallel recordings from intact strips and mucosa-free (detrusor) strips were performed so that the impact of the mucosa could be assessed.

### Neurogenic contractions and radiation

Frequency-dependent neurogenic contractions in intact bladder strips were negatively impacted by irradiation. Interestingly, this effect was absent in mucosa-free strips, consistent with the findings of Vale et al [24] in rat detrusor strips 6-months post-irradiation, suggesting that radiation did not directly damage intramural nerves, pre- or post-synaptic receptors or the contractile ability of smooth muscle cells. Given that neurogenic contractions were similar in control intact and mucosa-free tissues, and radiation only impacted neurogenic contractions in intact strips, it can be deduced that radiation affects signalling mechanisms in the mucosa. Pharmacological experiments where the radiation effect on neurogenic contractions in intact strips persisted during atropine but was reduced by the purinergic inhibitor PPADs suggested that purinergic mechanisms modulating neurogenic contractions are radiation-sensitive. The atropine-sensitive component (22%) and PPADs-effect here was similar to that reported by Kennedy et al [25] in guinea-pig bladder intact strips (28% atropine-sensitive, 50–60% reduction of atropine-resistant component by PPADs) who concluded that a non-cholinergic, non-P2X1-mediated component of neurogenic contractions was present. In the present study, the latter component was unaffected by radiation. The relative proportions of cholinergic and purinergic components were altered after irradiation (cholinergic 22% to 31% and purinergic 40% to 32%); such changes have been reported in animal models and human bladder samples of bladder dysfunction, some with enhanced cholinergic and others with enhanced purinergic components [26].

Neurogenic contractions in mucosa-free detrusor strips were similar to those in intact strips, indicating that normally, there is little input from mucosal cells to neurogenic contractions. Radiation may therefore be activating a mucosal mechanism that does not normally affect detrusor neurogenic contractions. Further work will determine whether irradiated tissues are more likely to release inhibitory factor(s) from mucosal cells such as the urothelium e.g. urothelium derived inhibitory factor [27] or interstitial cells upon EFS.

### Cholinergic, purinergic and receptor-independent contractions

The radiation-effect on neurogenic contractions in intact strips could also be explained by changes to pre-synaptic or post-synaptic mechanisms however, carbachol-evoked and ATP-evoked responses in intact or mucosa-free strips were unaffected by irradiation. This suggests that receptor expression and activity was not impaired by the radiation dose over the experimental time course. Intriguingly, irradiated mucosa-free strips exhibited larger agonist-evoked contractions than irradiated intact strips showing that the presence of the mucosal layer may have acted in some way to dampen the response. Given that carbachol- or ATP-contractions were similar in non-irradiated intact and mucosa-free strips, it seems likely that radiation activated an as yet unidentified mucosal mechanism that then modified the overall response to carbachol or ATP. Vale et al [24] reported increased purinergic sensitivity (application of α,β-methylene ATP) of detrusor smooth muscle from rats 6-month post-irradiation (15Gy or 25Gy) perhaps indicative of a time-dependent development of radiation-evoked purinergic mechanisms. The finding here that receptor-independent contractions (high-K^+^) were not affected by radiation nor by removal of the mucosal layer demonstrates that the ability of bladder smooth muscle to contract was radiation-resistant. Radiation may therefore have activated inhibitory mechanisms in the mucosal layer [27] that acted to dampen agonist-evoked and therefore receptor-mediated, post-synaptic mechanisms in the underlying detrusor while not affecting the contractile machinery of the smooth muscle.

### Spontaneous activity, radiation and the mucosa

Spontaneous activity in intact or mucosa-free bladder strips was not significantly impacted by irradiation. In non-irradiated strips, absence of the mucosa significantly reduced spontaneous activity, consistent with a positive mucosal effect, similar to the findings of others [16,28]. Several studies report spontaneous activity in mucosa-free strips [29–31] so while it seems that the detrusor layer is capable of generating spontaneous contractions, it is increasingly clear that this activity in intact strips is in some way modified or influenced by mucosal cells which include urothelial cells and interstitial cells in the lamina propria [32]. In the present study, irradiation apparently eliminated the mucosal effect however this needs further work as there was a trend towards reduced activity in irradiated strips where the mucosa was removed.

### Radiation and Ca^2+^-signalling in SMC

Spontaneous Ca^2+^-transients in SMC were observed in guinea-pig bladder preparations consistent with other reports [30]. SMC Ca^2+^-activity was reduced by irradiation, but this was apparently insufficient to significantly impact spontaneous contractions. While Ca^2+^-activity, particularly high-magnitude, long-duration and whole smooth muscle bundle Ca^2+^-flashes [30] is normally correlated with spontaneous contractions, the less-frequent, lower-amplitude Ca^2+^-events observed here in irradiated SMC here may have been sufficient to drive spontaneous activity. Gap junctions mediate intercellular communication in bladder smooth muscle; interestingly gap junction signaling is reportedly enhanced after radiation and is a primary mediator of radiation-bystander responses [4]. Further studies will ascertain whether radiation-enhanced gap junction signaling is involved in maintaining spontaneous contractions despite altered Ca^2+^-signaling within the acute timeframe of the present study.

A key strength of the study lies in the experimental design, enabling us to extend the early work on sub-acute radiation-effects and informing future *in vivo* research models of acute RIBT where additional mechanisms relating to urothelium loss and an inflammatory microenvironment complicate interpretation of contractility data. The findings of the present study demonstrate direct effects of irradiation on bladder strip contractility and this should now be studied in a longitudinal *in vivo* study to correlate early and late changes in physiology with development of inflammation and fibrosis. A limitation of interpretation is that this design does not directly model bladder irradiation in patients which is typically delivered as fractionated doses via external beams in intensity modulated radiation therapy (IMRT); however, an important consideration is brachytherapy where radiation emitting seeds are implanted e.g. to the prostate or cervix and are in close proximity to the bladder tissue.

## Conclusions

Our novel findings demonstrate that radiation targets the regulatory signalling mechanisms of the bladder mucosal layer, resulting in impaired neurogenic and enhanced agonist-evoked contractions and a concomitant reduction of Ca^2+^-transients in SMC. Furthermore, the important modulatory influence of the mucosal layer on spontaneous contractions in normal guinea-pig bladder tissues was confirmed and this was apparently eliminated by irradiation. Whether these changes persist through the acute phase of RIBT remains to be seen, however, the present results suggest that relief of RIBT symptoms and enhancement of the health-related quality-of-life of patients may result from the development of mucosal specific radioprotectant agents.
